# Perceived glucose levels matter more than CGM-based data in predicting diabetes distress in type 1 or type 2 diabetes: a precision mental health approach using *n*-of-1 analyses

**DOI:** 10.1007/s00125-024-06239-9

**Published:** 2024-07-30

**Authors:** Dominic Ehrmann, Norbert Hermanns, Andreas Schmitt, Laura Klinker, Thomas Haak, Bernhard Kulzer

**Affiliations:** 1 Research Institute Diabetes Academy Mergentheim (FIDAM), Bad Mergentheim, Germany; 2 Department of Clinical Psychology and Psychotherapy, University of Bamberg, Bamberg, Germany; 3 German Centre for Diabetes Research (DZD), München-Neuherberg, Germany; 4 Diabetes Clinic, Diabetes Centre Mergentheim (DZM), Bad Mergentheim, Germany

**Keywords:** Continuous glucose monitoring, Diabetes distress, Ecological momentary assessment, Precision mental health, Precision monitoring

## Abstract

**Aims/hypothesis:**

Diabetes distress is one of the most frequent mental health issues identified in people with type 1 and type 2 diabetes. Little is known about the role of glucose control as a potential contributor to diabetes distress and whether the subjective perception of glucose control or the objective glycaemic parameters are more important for the experience. With the emergence of continuous glucose monitoring (CGM), this is a relevant question as glucose values are now visible in real-time. We employed a precision monitoring approach to analyse the independent associations of perceived and measured glucose control with diabetes distress on a daily basis. By using *n*-of-1 analyses, we aimed to identify individual contributors to diabetes distress per person and analyse the associations of these individual contributors with mental health at a 3 month follow-up.

**Methods:**

In this prospective, observational study, perceived (hypoglycaemia/hyperglycaemia/glucose variability burden) and measured glucose control (time in hypoglycaemia and hyperglycaemia, CV) were assessed daily for 17 days using an ecological momentary assessment (EMA) approach with a special EMA app and CGM, respectively. Mixed-effect regression analysis was performed, with daily diabetes distress as the dependent variable and daily perceived and CGM-measured metrics of glucose control as random factors. Individual regression coefficients of daily distress with perceived and CGM-measured metrics were correlated with levels of psychosocial well-being at a 3 month follow-up.

**Results:**

Data from 379 participants were analysed (50.9% type 1 diabetes; 49.6% female). Perceived glucose variability (*t*=14.360; *p*<0.0001) and perceived hyperglycaemia (*t*=13.637; *p*<0.0001) were the strongest predictors of daily diabetes distress, while CGM-based glucose variability was not significantly associated (*t*=1.070; *p*=0.285). There was great heterogeneity between individuals in the associations of perceived and measured glucose parameters with diabetes distress. Individuals with a stronger association between perceived glucose control and daily distress had more depressive symptoms (β=0.32), diabetes distress (β=0.39) and hypoglycaemia fear (β=0.34) at follow-up (all *p*<0.001). Individuals with a stronger association between CGM-measured glucose control and daily distress had higher levels of psychosocial well-being at follow-up (depressive symptoms: β=−0.31; diabetes distress: β=−0.33; hypoglycaemia fear: β=−0.27; all *p*<0.001) but also higher HbA_1c_ (β=0.12; *p*<0.05).

**Conclusions/interpretation:**

Overall, subjective perceptions of glucose seem to be more influential on diabetes distress than objective CGM parameters of glycaemic control. *N*-of-1 analyses showed that CGM-measured and perceived glucose control had differential associations with diabetes distress and psychosocial well-being 3 months later. The results highlight the need to understand the individual drivers of diabetes distress to develop personalised interventions within a precision mental health approach.

**Graphical Abstract:**

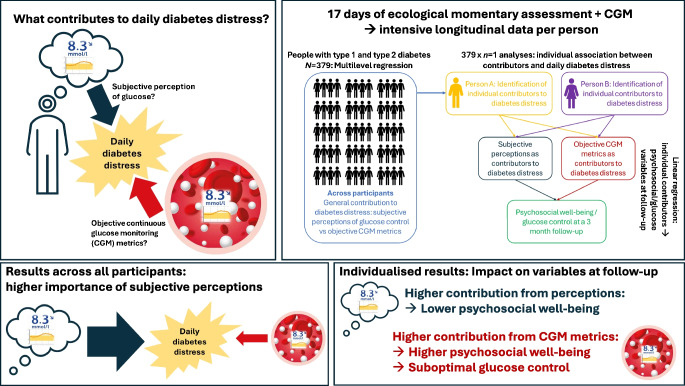

**Supplementary Information:**

The online version of this article (10.1007/s00125-024-06239-9) contains peer-reviewed but unedited supplementary material.


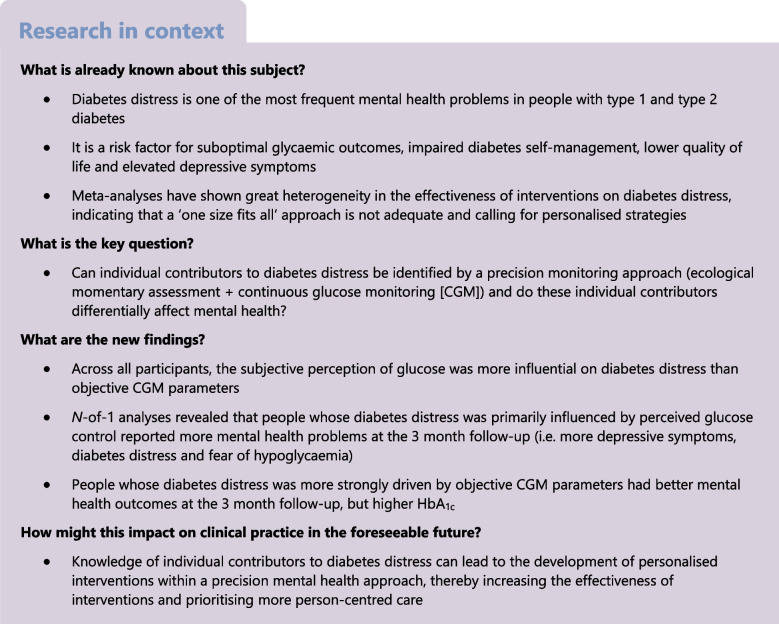


## Introduction

Diabetes management, achieving optimal glycaemic control and avoiding acute and long-term complications are largely dependent on the self-management of people with diabetes [1–3]. For optimal diabetes self-management, specific knowledge and skills are essential; however, to keep glucose levels in an optimal range, consistent motivation and emotional stability to conduct multiple self-management tasks on a day-to-day basis are equally important [1, 3]. Recently, perspective was gained on the considerable self-management strain brought by diabetes, as evidence found that people with diabetes spend an average of 77 min per day thinking about their diabetes [4]. This constant need for daily self-management and glucose control can thus be a significant burden and lead to emotional distress [5–7].

The negative emotional side of living with diabetes is referred to as diabetes distress, which includes worries, concerns, fears and perceived threats associated with diabetes, its therapy and the associated acute and long-term complications [6, 7]. Research conducted over the last 30 years has established diabetes distress as a clinically important risk factor for suboptimal health outcomes in people with type 1 and type 2 diabetes [5, 8]. Previous research has demonstrated that elevated diabetes distress is associated with biological markers, such as higher HbA_1c_ [9–11] and lower heart rate variability [12]. Additionally, there is evidence that psychosocial distress is associated with a higher mortality rate [13]. Further, it has been shown that elevated diabetes distress is associated with reduced self-care activities and impaired diabetes self-management [14, 15], lower quality of life [5, 8] and more depressive symptoms [8, 16, 17].

Elevated diabetes distress is quite common, with a prevalence of 30–40% [18, 19]. It has also been shown that a certain chronicity of elevated distress must be assumed in approximately 74% of people affected by elevated diabetes distress [6]. Using ambulatory assessment, we demonstrated that people with type 1 diabetes spend nearly 24% of their days with elevated diabetes distress, translating to nearly 1 week per month [20].

Therefore, interventional strategies to reduce diabetes distress are of great clinical importance [6, 21]. Although different psychological interventions aiming to reduce diabetes distress have been developed and tested in trials, there is a large heterogeneity in the found effectiveness, as shown by the results of relevant meta-analyses [22–25]. In the meta-analysis by Sturt et al, the standardised mean difference (SMD) in ten out of 11 studies did not reach significance, while the overall SMD was significant [22]. In a meta-analysis of acceptance–commitment therapy interventions, the SMD in three out of four trials was not significant [23]. Similarly, in a recent meta-analysis by Jenkinson et al, the SMD in 18 out of 23 trials on cognitive behavioural therapy was not significant, with one study even favouring the control condition [24]. Heterogeneity was also found in the meta-analysis by Schmidt et al, where Cohen’s *d* ranged from 0.05 to 1.30 [25].

This heterogeneity in effectiveness at reducing diabetes distress may indicate that a ‘one size fits all’ approach is not appropriate, and that individualised strategies are needed. This aligns with a recent joint consensus report from the ADA and the EASD calling for the implementation of personalised, precision medicine approaches in diabetes care [26]. To address diabetes distress in a personalised manner, the individual aetiology of diabetes distress must be understood. However, as Skinner et al highlighted in their review, there is an abundant lack of data on the aetiology and development of diabetes distress [5].

Therefore, in our study, we implemented a precision monitoring approach and combined continuous glucose monitoring (CGM) and ecological momentary assessment (EMA) [27]. EMA is a methodology that allows the repeated sampling of people’s experiences and feelings in real-time in an ambulatory, real-world setting [28]. Thus, by combining data from CGM and EMA, we were able to map diabetes distress on a daily basis and analyse associations of daily diabetes distress with CGM-assessed glucose levels and subjective perceptions on a day-to-day basis.

In particular, we wanted to analyse whether objective CGM-based glycaemic parameters on a given day were associated with the experience of diabetes distress on that day or whether the subjective perceptions of hypoglycaemia, hyperglycaemia and glucose variability would play a greater role. In doing so, we aimed to identify potential individual glycaemic contributors to diabetes distress.

Based on these individual contributors or drivers of diabetes distress, we aimed to achieve a precision mental health approach. The approach of CGM and EMA over several days enabled the sampling of intensive longitudinal data that allowed for the conducting of *n*-of-1 analyses. We sought to quantify the individual impacts of objective CGM-based and subjective glucose perceptions on daily diabetes distress for each person, and to assess how these individual drivers of diabetes distress affect future psychosocial and glucose outcomes in people with type 1 and type 2 diabetes. This aimed at finding starting points for the conceptualisation of personalised and more effective interventions for diabetes distress following a precision mental health approach.

In summary, the present analysis had two aims: (1) to determine whether objective CGM-based glycaemic parameters (time in hypoglycaemia, time in hyperglycaemia and glucose variability) or subjective perceptions of hypoglycaemia, hyperglycaemia and glucose variability show stronger associations with daily diabetes distress; and (2) to determine these associations for each person and assess whether these individualised contributors (objective vs subjective) have a differential predictive impact on future psychosocial and glycaemic outcomes.

## Methods

This analysis involved two prospective, observational, non-interventional studies on people with type 1 diabetes and type 2 diabetes: the DIA-LINK1 study (ClinicalTrials.gov registration no. NCT03811132) and the DIA-LINK2 study (ClinicalTrials.gov registration no. NCT04438018), respectively. Ethical approval was obtained from the German Psychological Society for both studies (DIA-LINK1: NH082018; DIA-LINK2: NH082018_2). The current analyses focused on the 17 day ambulatory assessment period after baseline, where diabetes distress was assessed daily, and the follow-up period, which took place 3 months after the baseline assessment. The DIA-LINK studies were identical in their conduct and in the variables assessed. In both studies, gender was considered as a factor that potentially influenced the experiencing or reporting of diabetes distress. Further information about the study design has been published elsewhere [12, 20].

### Participants

Recruitment for both studies took place at the Diabetes Clinic Mergentheim, a specialised inpatient diabetes centre in Germany. Reasons for admission into our centre include sustained hyperglycaemia, occurrence of complications or psychosocial issues complicating the treatment and course of diabetes. Patients who were admitted due to diabetic emergencies (ketoacidosis or severe hypoglycaemia) were not recruited. Participants for DIA-LINK1 were recruited between March 2019 and March 2020; for DIA-LINK2, recruitment took place between July 2020 and December 2021. In each of the studies, the recruitment target was set at *N*=200.

The following inclusion criteria were applied: type 1 diabetes (only DIA-LINK1) or type 2 diabetes (only DIA-LINK2), diabetes duration ≥1 year, age between 18 and 70 years, sufficient German language skills, smartphone compatible with the EMA app and informed consent. The following exclusion criteria were applied: inability to consent, illness with significant impairment of cognitive function (e.g. dementia), severe somatic illness or mental disorder that may interfere with study participation, terminal illness or being bedridden.

Trained study personnel informed participants about the study, both orally and in writing. Written informed consent was obtained from all participants prior to study inclusion.

### Study conduct

After inclusion, participants’ demographic and medical data were collected from medical records or via case report forms (baseline assessment). Participants without a CGM device were equipped with an unblinded, intermittently scanned CGM system (isCGM; FreeStyle Libre 2, Abbott Diabetes Care, Germany) for the assessment periods. For the EMA, participants’ personal smartphones were used and a smartphone app (‘mEMA’, Ilumivu Software for Humanity, Asheville, NC, USA) that contained the EMA protocol installed. To ensure proper functioning, the EMA procedure was tested during participants’ inpatient stay. The actual EMA period started in an ambulatory setting in participants’ everyday life, beginning on the first Saturday after discharge from the hospital. EMA questions were prompted daily over 17 consecutive days. Three months after baseline, a follow-up assessment took place, with participants completing questionnaires and with the collection of CGM data over a 14 day period.

### Assessments

The key variable of interest was daily diabetes distress, which was assessed via EMA for 17 consecutive days using five questions from the Problem Areas in Diabetes (PAID) questionnaire, adapted for the daily assessment (e.g. How much did you feel overwhelmed by your diabetes today? How much has diabetes treatment taken away your mental/physical strength today? How much did you feel left alone in your diabetes treatment today?) [7, 20]. Furthermore, participants were asked daily about the subjectively perceived glucose-specific burden (burden due to hypoglycaemia [<3.9 mmol/l], hyperglycaemia [>10 mmol/l] and glucose variability). All EMA questions were transformed to a scale range of 0–100, where higher scores indicate higher burden. The reliability and validity of the five daily diabetes distress questions and the three daily glucose-specific burden measures have been established [20]. Participants received prompts, on the EMA app, to answer the questions every evening. During the EMA period, objective CGM-based glucose parameters were determined for each day separately: time in hypoglycaemic range (percentage <3.9 mmol/l), time in hyperglycaemic range (percentage >10 mmol/l) and glucose variability as CV.

At baseline and the 3 month follow-up, participants completed the following questionnaires:

- Diabetes distress: The PAID questionnaire consists of 20 items assessing emotional problems and diabetes-specific burdens. A total score was derived and transformed to a scale from 0 to 100, with higher values indicating higher diabetes distress [7].
- Depressive symptoms: The Centre for Epidemiological Studies Depression (CES-D) scale contains 20 items to assess the frequency of depressive symptoms over the previous week [29]. A total score was calculated, ranging from 0 to 60, with higher values indicating stronger depressive symptoms. Incidence and persistence of depressive symptoms can be calculated using a cut-off score of 22.
- Fear of hypoglycaemia: The short form of the Hypoglycaemia Fear Survey (HFS-II) was used [30]. A sum score for the total scale was calculated, with higher scores indicating greater fear of hypoglycaemia.
- Fear of diabetes complications: A short form of the Fear of Diabetes Complications Questionnaire (FDCQ-SF) was used, containing six items [31]. A sum score was calculated, with higher scores indicating greater fear of complications.
- Diabetes self-management: The revised Diabetes Self-Management Questionnaire (DSMQ-R), containing 27 items assessing different aspects of daily self-management, was used [32]. A sum score was calculated, with higher values indicating more optimal self-management behaviours.
- Diabetes acceptance: The Diabetes Acceptance Scale (DAS) assesses the acceptance and integration of diabetes into daily life [33]. A sum score was derived, with higher scores indicating greater acceptance.

HbA_1c_ was measured in a central laboratory (Automated Glycohemoglobin, Analyzer HLC-723G11; Tosoh Europe, Belgium) (normal range 21–43 mmol/mol [4.1%–6.1%]) at baseline and at the 3 month follow-up. During the follow-up phase, CGM-based glucose parameters were calculated for the 14 day period. Demographic information was collected via medical chart review or via self-report. Gender was assessed via self-report. Ethnicity was self-reported by asking about country of birth.

### Statistical analyses

To analyse whether objective CGM-based glucose parameters or subjectively experienced glucose distress was more strongly associated with daily diabetes distress, a Bayesian linear mixed-effects regression analysis was conducted. This analysis considered the repeated daily observations for each participant (i.e. nested design). The dependent variable was EMA-based diabetes distress on each day. Independent variables of interest were markers of subjectively perceived glucose control, namely burden due to hypoglycaemia, hyperglycaemia and glucose variability. Further independent variables of interest were markers of objective CGM-measured indicators of glucose control, specifically percentage of glucose values <3.9 mmol/l, percentage of glucose values >10 mmol/l and glucose CV. All independent variables of interest were included in the model at the day level (one value per variable per day per participant) and as random factors. The analysis was controlled for gender, type of diabetes, prior CGM use (yes/no), number of late complications and study day. Sensitivity analyses were performed, where the analysis was conducted separately for people with type 1 and type 2 diabetes, and for people with and without CGM experience.

The inclusion of subjective and objective markers of glucose management as random factors allowed for the calculation of individual estimates of the association between each independent variable and daily diabetes distress per person (i.e. *n*-of-1 analyses). Thus, for each person, individual associations between diabetes distress and all perceived and CGM-measured markers of glucose were extracted. Associations of these individual subjective and objective contributors to diabetes distress with psychosocial and glucose parameters at follow-up were investigated using linear and logistic regression analysis. For these analyses, the dependent variable was the respective questionnaire score or glucose parameter at follow-up, with the individual associations as independent variables, controlled for gender, age and type of diabetes. The aim of this analysis was to investigate whether there is a differential impact on follow-up variables based on diabetes distress being primarily driven by subjective perceptions of glucose control or objective CGM metrics.

A sensitivity analysis was performed to ascertain whether meaningful associations remained when the respective subjective and objective markers were averaged to obtain two overall scores of possible drivers of diabetes distress. In doing so, the mean of the associations (Fisher *Z*-transformed) of the three subjective glucose markers (burden due to hypoglycaemia, hyperglycaemia and glucose variability) was calculated per person. This served as an overall indicator of subjective glucose control as a contributor to diabetes distress and indicated the extent to which diabetes distress in a specific person arises from subjectively perceived glucose control. Similarly, the mean of associations (Fisher *Z*-transformed) of the three objective glucose markers (percentage <3.9 mmol/l, percentage >10 mmol/l, CV) was calculated. This served as an overall indicator of objective glucose management as a contributor to diabetes distress and indicated the extent to which diabetes distress in a specific person arises from their objective CGM-based glucose control. All statistical analyses were performed with R version 4.2.3 (package blme, version 1.0-5: https://cran.r-project.org/web/packages/blme/index.html) and JASP version 0.18.1 (https://jasp-stats.org/).

## Results

### Baseline characteristics

For the current analysis, the data of 379 participants were analysed, including 193 people with type 1 diabetes (DIA-LINK1) and 186 people with type 2 diabetes (DIA-LINK2). Sample characteristics are displayed in Table 1.

**Table 1:** Sample characteristics

Characteristic (total *N*=379)	DIA-LINK1: type 1 diabetes	DIA-LINK2: type 2 diabetes
*n*	193 (50.9)	186 (49.1)
Age (years)	39.1±12.7	52.8±9.7
Gender		
Male	80 (41.5)	111 (59.7)
Female	113 (58.5)	75 (40.3)
BMI (kg/m^2^)	26.3±5.2	35.5±7.0
Country of birth		
Germany	176 (91.2)	170 (91.4)
Other European countries	10 (5.2)	8 (4.3)
Central Asia	6 (3.1)	6 (3.2)
Africa	1 (0.5)	0 (0.0)
North America	0 (0.0)	2 (1.1)
Years of education	13.2±2.6	12.6±2.6
Duration of diabetes (years)	18.9±11.9	11.9±7.6
HbA_1c_ (%)	8.6±1.9	9.0±1.7
HbA_1c_ (mmol/mol)	70±21	75±19
Diabetes treatment regimen		
Non-insulin therapy	0 (0.0)	27 (14.5)
Simple insulin regimen (e.g. basal insulin therapy)	0 (0.0)	136 (73.1)
Multiple daily insulin injections	82 (42.5)	22 (11.8)
Insulin pump therapy	111 (57.5)	1 (0.5)
Long-term complications^a^ (mean per person)	0.69±0.85	1.22±1.41
Psychosocial well-being (baseline assessment + 17 day EMA phase)		
Diabetes distress (PAID sum score, range: 0–100)	39.8±17.9	42.0±18.9
Depressive symptoms (CES-D sum score, range: 0–60)	21.2±11.5	21.9±11.8
Time with diabetes distress (%)^b^	22.1±27.9	23.9±31.7
Time with hypoglycaemia distress (%)^b^	23.0±19.3	12.8±18.9
Time with hyperglycaemia distress (%)^b^	56.5±26.3	43.1±32.2
Time with glucose variability distress (%)^b^	46.0±27.2	37.3±30.9
CGM-derived parameters of glucose control (17 day EMA phase)		
Mean glucose (mmol/l)	9.8±2.1	8.49±2.2
Percentage <3.9 mmol/l	3.8±3.9	1.2±2.3
Percentage 3.9–10 mmol/l	54.5±17.0	74.9±22.7
Percentage >10 mmol/l	41.7±18.5	23.9±23.1
Glucose fluctuations (CV)	32.2±4.9	22.2±5.1

Data are reported as mean±SD or *n* (%)

^a^List of complications: retinopathy, neuropathy, nephropathy, diabetic foot syndrome, cardiovascular disease, apoplexy, arterial vascular disease

^b^Calculated as the percentage of days with elevated distress

Notably, people with type 1 or type 2 diabetes (50.9% type 1 diabetes, 49.6% female) reported considerable time with distress due to glucose variability (46.0% and 37.3%, respectively), despite rather low actual glucose variability (32.2% and 22.2%, respectively) (Table 1). This translates into 6 and 5 days of the 17 day EMA period that were spent with elevated distress due to glucose variability for people with type 1 and type 2 diabetes, respectively.

### Glucose-specific drivers of diabetes distress

**Fig. 1:**
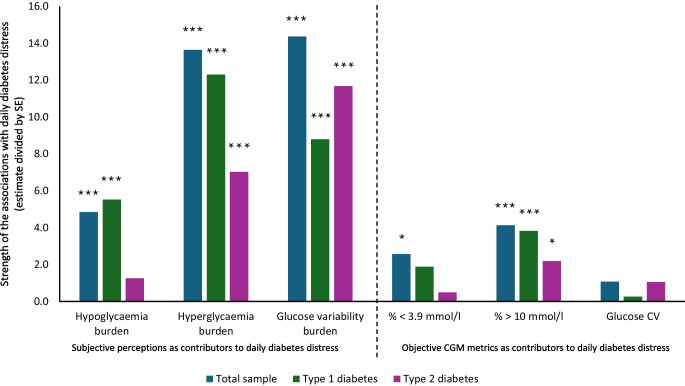
Strength of the associations with daily diabetes distress of subjective perceptions of glucose control vs objective CGM-based metrics of glucose control. SE, SEM. *p<0.05; ***p<0.001

The mixed-effects linear regression revealed that both subjective perception of glucose control and objective CGM-based metrics of glucose control were significantly associated with diabetes distress on a daily level (Table 2, Fig. 1). The subjective perception of glucose variability was numerically the strongest predictor of daily diabetes distress (*t*=14.360; *p*<0.0001), controlled for the CGM-based variability. In contrast, CGM-based glucose variability (CV) was not significantly associated with daily diabetes distress (*t*=1.070; *p*=0.285). Overall, associations with the subjective perceptions of glucose control were stronger than associations with the objective CGM metrics. Increases of 10% in burden due to hyperglycaemia (*t*=13.637; *p*<0.0001) and glucose variability were associated with 1.43% and 1.75% increases in daily diabetes distress, respectively (Table 2). In contrast, increases of 10% in time in hypoglycaemia (percentage <3.9 mmol/l; *t*=2.561; *p*=0.0105) and hyperglycaemia (percentage >10 mmol/l; *t*=4.126; *p*<0.0001) were associated with 0.93% and 0.52% increases in daily diabetes distress, respectively. Women and people with type 2 diabetes reported significantly more daily diabetes distress, while prior CGM use and number of late complications did not show significant associations (Table 2). Zero-order correlations indicating raw associations between perceived and measured glucose control are shown in electronic supplementary material (ESM) Table 1.

**Table 2:** Subjective perceptions vs objectively measured glucose control as drivers of daily diabetes distress

Variable	Estimate	SEM	*t* value	*p* value
Subjective perceptions of glucose control as contributors to diabetes distress				
Hypoglycaemia burden	0.053	0.011	4.844	<0.0001
Hyperglycaemia burden	0.143	0.011	13.637	<0.0001
Glucose variability burden	0.175	0.012	14.360	<0.0001
Objective CGM-based markers of glucose control as contributors to diabetes distress				
Percentage <3.9 mmol/l	0.093	0.036	2.561	0.0105
Percentage >10 mmol/l	0.052	0.013	4.126	<0.0001
Glucose CV	−0.027	0.025	1.070	0.285

Controlled for gender (estimate=0.033, *p*=0.012), type of diabetes (estimate=0.050, *p*=0.0009), prior CGM use (estimate=−0.026, *p*=0.075), number of late complications (estimate=−0.008, *p*=0.151) and study day (estimate=0.0003, *p*=0.385)

Estimates were from the Bayesian mixed-effects linear regression analysis with participant as nested variable. The dependent variable was daily diabetes distress. Independent variables were included simultaneously in the model and defined as random effects

Separate analyses for type of diabetes revealed that for people with type 2 diabetes, the perception of glucose variability was a more influential factor for diabetes distress than for people with type 1 diabetes (Fig. 1, ESM Table 2). However, for people with type 1 diabetes, the perception of hypoglycaemia was more influential than for people with type 2 diabetes (Fig. 1, ESM Table 2). A highly comparable pattern of associations was found for people with vs without prior CGM use (ESM Table 3). For people without prior CGM use, the association with time in hypoglycaemia was significant; this was not the case for people with prior CGM use (ESM Table 3).

### *N*-of-1 analyses

Cumulative distribution of individual associations showed a rather wide range across all participants (ESM Fig. 1). For example, a 10-point increase in burden due to variability was associated with an increase of more than 2% in diabetes distress for 20% of the participants (ESM Fig. 2c). In some participants, negative associations with daily diabetes distress were found for both subjective (ESM Fig. 2a–c) and objective (ESM Fig. 2d–f) markers of glucose control. This indicates that increased burden due to hypoglycaemia was associated with lower daily diabetes distress for some participants (ESM Fig. 2a) or that more time in hyperglycaemia was associated with lower daily diabetes distress (ESM Fig. 2e).

### Impact of individual associations on psychosocial and glucose parameters at 3 month follow-up

**Fig. 2:**
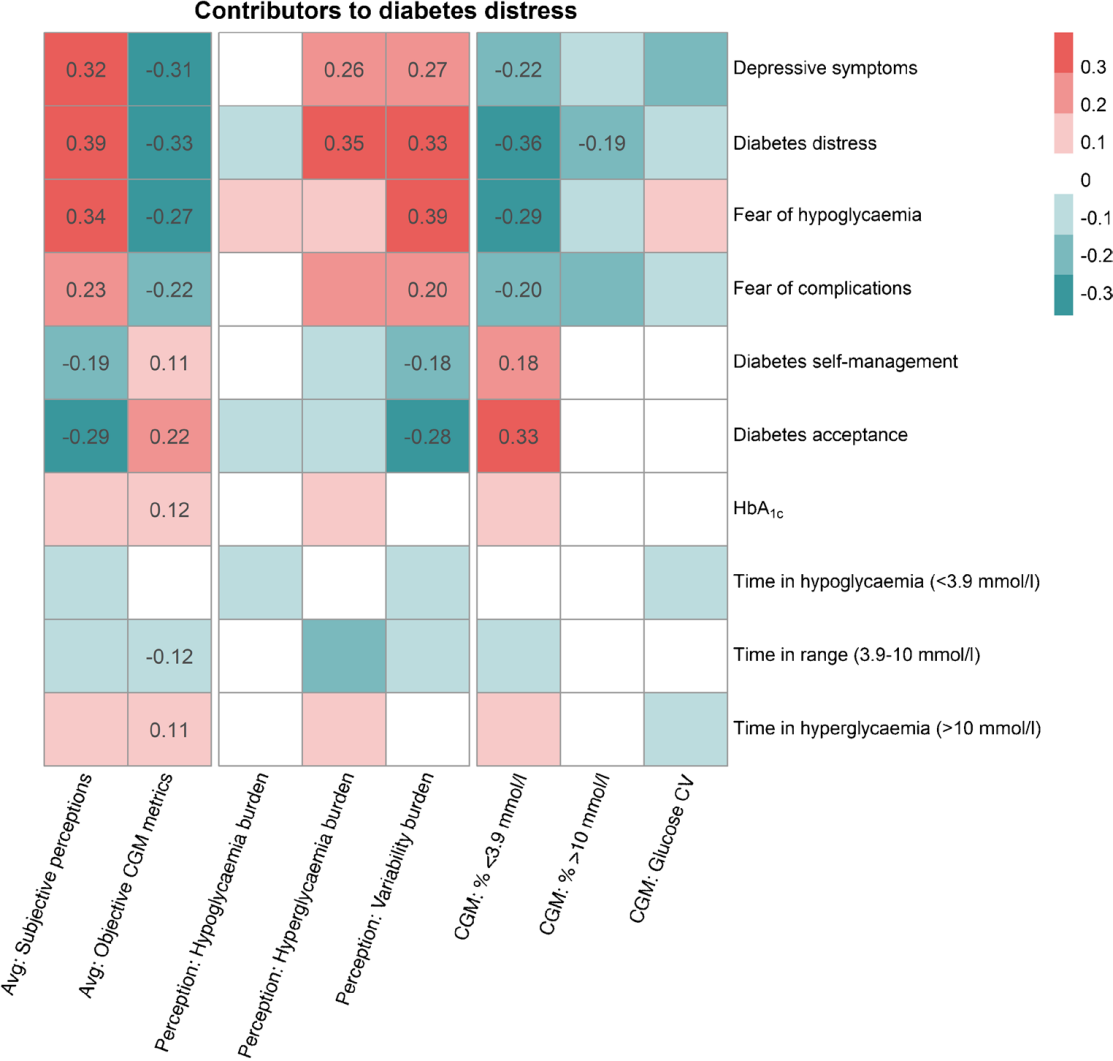
Heatmap of the associations of the glucose-specific drivers of diabetes distress (columns) with psychosocial and glucose parameters at the 3 month follow-up (rows). Depicted are the results from separate linear regression analyses: dependent variables can be seen on the right side, indicating the rows of the heatmap. Independent variables were the individual associations from *n*-of-1 analyses between daily diabetes distress and the respective variable at the bottom of the heatmap. The independent variables in the columns of the heatmap depict the individual contributors to diabetes distress during the EMA phase. ‘Avg: Subjective perceptions’ represents the individual associations of the mean of hypoglycaemia burden, hyperglycaemia burden and variability burden with daily diabetes distress. This indicates the strength of overall subjective perceptions of glucose control as a contributor to daily diabetes distress. ‘Avg: Objective CGM metrics’ represents the individual associations of the mean of CGM-based percentage <3.9 mmol/l, percentage >10 mmol/l and glucose CV with daily diabetes distress. This indicates the strength of overall objective CGM metrics of glucose control as a contributor to daily diabetes distress. ‘Perception: XYZ’ represents the individual associations of the respective perception of glucose control with daily diabetes distress. This indicates the strength of this perception as a contributor to daily diabetes distress. ‘CGM: XYZ’ represents the individual associations of the respective CGM metric of glucose control with daily diabetes distress. This indicates the strength of this CGM metric as a contributor to daily diabetes distress. The colour palette indicates the standardised regression coefficients, with darker colours representing larger coefficients (see legend). Only significant (*p*<0.05) associations are displayed. Linear regression analyses were controlled for gender, age and type of diabetes

The individual associations of subjective and objective markers of glucose management with daily diabetes distress indicate the extent to which perceived and CGM-based glucose control contribute to each person’s diabetes distress. The strength of perceived and CGM-based contributors of diabetes distress showed significant and differential associations with psychosocial variables at follow-up (Table 3). Figure 2 shows a heatmap of the associations between the different glucose-specific contributors (i.e. contributors to daily diabetes distress) and psychosocial well-being and glucose control at the 3 month follow-up. Individuals with a greater association between subjective variability burden and diabetes distress during the EMA phase (i.e. variability burden as a greater contributor to diabetes distress) had more depressive symptoms (*p*<0.001), greater diabetes distress (*p*<0.001), higher fear of hypoglycaemia (*p*<0.001), more fear of complications (*p*=0.004), suboptimal diabetes self-management (*p*=0.005) and lower diabetes acceptance (*p*<0.001) at follow-up. Similarly, where hyperglycaemia burden was a greater contributor to diabetes distress (i.e. stronger associations), depressive symptoms were greater (*p*=0.020) at the follow-up. In contrast, individuals for whom CGM-based time in hypoglycaemia was a stronger contributor to diabetes distress showed fewer depressive symptoms (*p*=0.007), less diabetes distress (*p*<0.001), less fear of hypoglycaemia (*p*<0.001), less fear of complications (*p*=0.022), more optimal diabetes self-management (*p*=0.020) and greater diabetes acceptance (*p*<0.001) at follow-up. Notably, no significant associations were found with glucose parameters at follow-up. However, in people with type 1 diabetes, individuals for whom hyperglycaemia burden was a greater contributor to diabetes distress showed higher HbA_1c_ levels (β=0.37; *p*=0.041), less time in range (β=−0.43; *p*=0.011) and more time in hyperglycaemia (β=0.42; *p*=0.015) at follow-up (ESM Table 4), in contrast to people with type 2 diabetes (ESM Table 5).

**Table 3:** Impact of individual associations of subjective and objective markers of glucose management on psychosocial and glucose parameters at follow-up

Variable at follow-up	Subjective perceptions as contributors to diabetes distress			Objective CGM metrics as contributors to diabetes distress		
	Hypoglycaemia burden	Hyperglycaemia burden	Variability burden	Percentage <3.9 mmol/l	Percentage >10 mmol/l	Glucose CV
Depressive symptoms	0.03	0.26*	0.27***	−0.22**	−0.14	−0.16
Diabetes distress	−0.06	0.35***	0.33***	−0.36***	−0.19*	−0.09
Fear of hypoglycaemia	0.07	0.12	0.39***	−0.29***	−0.13	0.07
Fear of complications	−0.02	0.20	0.20**	−0.20*	−0.15	−0.06
Diabetes self-management	−0.01	−0.11	−0.18**	0.18*	−0.01	−0.01
Diabetes acceptance	−0.04	−0.14	−0.28***	0.33***	−0.01	−0.03
HbA_1c_	0.01	0.15	0.00	0.10	0.04	−0.03
Time in hypoglycaemia (<3.9 mmol/l)	−0.04	−0.03	−0.05	0.02	−0.01	−0.04
Time in range (3.9–10 mmol/l)	0.04	−0.15	−0.04	−0.13	−0.03	0.06
Time in hyperglycaemia (>10 mmol/l)	−0.03	0.15	0.04	0.12	0.03	−0.05

Data are standardised regression coefficients from linear regression analysis

Controlled for gender, age and type of diabetes

^*^p<0.05, **p<0.01, ***p<0.001

### Sensitivity analysis: overall subjective vs objective contributors to diabetes distress

As can be seen in Fig. 2, significant correlations with psychosocial variables remained in the analysis of the mean of the associations of subjective markers of glucose control (i.e. subjective contributor to diabetes distress) (see also ESM Table 6). A greater contribution of overall subjective perceptions of glucose control to diabetes distress during the EMA phase was associated with lower levels of psychosocial well-being at follow-up (Fig. 2; e.g. more depressive symptoms [β=0.32], diabetes distress [β=0.39] and hypoglycaemia fear [β=0.34]; all *p*<0.001). The mean of associations of objective CGM markers (i.e. objective contributor to diabetes distress) showed significant associations with higher levels of psychosocial well-being at follow-up (Fig. 2; e.g. less depressive symptoms [β=−0.31], diabetes distress [β=−0.33], hypoglycaemia fear [β=−0.27]; all *p*<0.001). In addition, a greater contribution of overall objective CGM metrics to diabetes distress during the EMA phase significantly correlated with higher HbA_1c_ (β=0.12; *p*=0.024), less time in range (β=−0.12; *p*=0.032) and more time in hyperglycaemia (β=0.11; *p*=0.037) at follow-up (Fig. 2, ESM Table 6). Furthermore, the differential impact of both contributors on incidence and remission of elevated depressive symptoms is depicted in ESM Fig. 2.

## Discussion

The analysis of glucose-specific contributors to diabetes distress demonstrated that subjective perceptions of hyperglycaemia and glucose variability were the most important glucose-related drivers of daily diabetes distress. Although objective CGM-based times in hypo- and hyperglycaemia were significantly associated with daily diabetes distress, the magnitude of associations was higher for subjective experiences. Interestingly, subjective perceptions of glucose variability were highly associated with daily diabetes distress, whereas actual variability according to CGM was not significantly associated. This is particularly important, as the subjective perceptions of hypoglycaemia, hyperglycaemia and glucose variability were controlled for the objective CGM-based exposure to hypo- and hyperglycaemia as well as glucose CV.

In addition, glucose CV was rather low in our sample, but participants felt considerably burdened by glucose variability. This suggests that the subjective interpretation of glucose fluctuations may be more relevant for the overall experience of diabetes as distressing than actual glucose variability. Interestingly, this seems to be even more the case for people with type 2 diabetes. Furthermore, subjective interpretation of glucose variability seems to be rather independent from the level of objective variability, which highlights the need to educate people with diabetes on how to interpret glucose fluctuations and to accept fluctuations as normal. Such education and normalisation may help with the perceived obtrusiveness of glucose fluctuations, as fluctuations may hamper daily activities and can therefore be perceived as an annoyance [34].

The importance of subjective perceptions of glucose variability is further supported by the significant associations with psychosocial variables at follow-up. These associations suggest that if the subjective perception of glucose variability is a major driver of diabetes distress, that perception may play a role in decreased levels of psychosocial well-being, suboptimal diabetes self-management and reduced diabetes acceptance. Further, the mean of individual associations of perceived and CGM-measured glucose control showed significant differential associations with psychosocial and glucose parameters at follow-up. For psychosocial well-being at follow-up, it was better when diabetes distress was informed by objective CGM metrics compared with subjective perceptions. In contrast, for glucose control at follow-up, it was more detrimental when diabetes distress was informed by objective CGM metrics. This pattern highlights the need to understand potential drivers of diabetes distress and their differential impacts. However, it is important to note that objective CGM metrics as drivers of diabetes distress are not beneficial per se, because while they appear to have a positive impact on psychosocial well-being, they appear to have a negative impact on glucose control.

The somewhat counter-intuitive finding that some individuals showed associations of more time in hyperglycaemia with lower diabetes distress underlines the relevance of understanding contributors to diabetes distress on an individual basis. Objective CGM-based glucose markers may also vary in subjective interpretation due to different psychosocial variables, for instance, levels of fear of hypoglycaemia or fear of complications. Higher glucose levels may be associated with less diabetes distress in people with greater hypoglycaemia fear [35]. Thus, in clinical practice, discussion of both CGM- and EMA-based diabetes distress data could lead to new insights and identification of potential mechanisms behind, for example, fear of hypo- or hyperglycaemia. The use of CGM and EMA has the potential to improve person-centred care by providing a systematic assessment of glucose and person-reported outcomes for discussion between people with diabetes and healthcare professionals.

To our knowledge, such individualised findings of the associations of potential drivers of diabetes distress have not been previously shown. The findings highlight that it is important to understand the potential contributors to diabetes distress in individuals as various contributors may have a differential impact on diabetes management. In order to acknowledge individual subjective interpretations in clinical practice, a person-centred approach addressing drivers of diabetes distress is recommended [6, 36]. Important practice and intervention implications could be drawn from understanding whether diabetes distress is informed by subjective perceptions and interpretations of glucose control or whether it is informed by objective CGM-based glucose parameters. For the former, the results suggest that it may be reasonable to shift the attention to the objective glucose values and discuss personal goals and attitudes regarding CGM-based glucose parameters. For the latter, therapy adjustments may affect the experience of diabetes distress.

The systematic assessment and addressing of individual drivers of diabetes distress has the potential to increase the effectiveness of interventions for diabetes distress [22–25]. However, to increase the feasibility of an EMA approach in a clinical setting, the merging of CGM and EMA data, statistical analysis and pattern recognition require automation. Such an approach could be implemented in digital health applications that automatically detect increased levels of diabetes distress, identify potential contributors and offer timely low-grade individualised interventional measures. Such just-in-time adaptive interventions have shown promising results [37, 38], but implementation in diabetes care is lacking thus far [39].

When interpreting the findings, the following limitations must be considered. First, the current EMA approach focused on glucose-related drivers of diabetes distress only. Thus, the EMA results cannot extend to other contributors that were not considered. Future research could also focus on the subdomains of diabetes distress [40, 41]. Second, participants were recruited from an inpatient setting and had suboptimal glucose control. Therefore, a selection bias cannot be excluded. However, the EMA period started after discharge from the hospital, in participants’ daily lives. Furthermore, due to the recruitment strategy, 50% of the sample had elevated diabetes distress or depressive symptoms. Thus, generalisability may be limited. Generalisability regarding gender can be assumed since an equal number of men and women participated and the analyses were controlled for the effect of gender on diabetes distress. Third, the repeated assessments may have induced some bias due to reactivity and consequential behavioural changes. Lastly, there is a conceptual and methodological overlap between the assessment of daily diabetes distress and the assessment of perceived glucose control, as both are subjective experiences. Therefore, a stronger association between these constructs might be expected. However, there are still significant associations between diabetes distress and CGM-based time in hypo- and hyperglycaemia, but not with CGM-based glucose CV. This suggests differential associations of the glucose parameters. The differential associations were further highlighted by the *n*-of-1 analyses, where the methodological overlap was less important, as the impact of perceived and measured glucose control on psychosocial well-being was analysed equally for every person.

In summary, the combination of EMA and CGM enabled identification of glucose-related drivers of individual diabetes distress. Subjective perceptions of glucose control, particularly burden due to hyperglycaemia and glucose variability, were more influential on diabetes distress than objective CGM parameters. The individual contributors showed differential associations, indicating that subjective perceptions as drivers of diabetes distress are associated with reduced levels of psychosocial well-being and higher risks for incident and persistent depressive symptoms, while objective contributors demonstrated diametric associations. The results regarding individual drivers of diabetes distress show the potentials and likely benefits of combining EMA and CGM within a precision mental health approach. Taken together, implementing such a precision mental health approach can help identify personalised starting points for interventional measures and increase person-centred care.

## Supplementary Information

Below is the link to the electronic supplementary material.

ESM (PDF 589 KB)

## Data Availability

The following data can be shared: individual participant data that underlie the results reported in this article, after de-identification (text, tables, figures and ESM). Also, the study protocol can be made available. Data sharing can commence immediately following publication until 10 years after publication. Data will be shared with researchers who provide a methodologically sound proposal. Sharing of the data must fulfil the purpose of achieving the aims in the approved proposal. Proposals should be directed to ehrmann@fidam.de. To gain access, data requestors will need to sign a data access agreement.

## Abbreviations

CES-D: Centre for Epidemiological Studies Depression
CGM: Continuous glucose monitoring
EMA: Ecological momentary assessment
PAID: Problem Areas in Diabetes
SMD: Standardised mean difference
