# Supplementary material for: Perceived glucose levels matter more than CGM-based data in predicting diabetes distress in type 1 or type 2 diabetes: a precision mental health approach using n-of-1 analyses
Source: Diabetologia. 2024 Jul 30;67(11):2433–45. doi: 10.1007/s00125-024-06239-9 (PMC11519212; doi:10.1007/s00125-024-06239-9)

Perceived glucose levels matter more than CGM-based data in predicting diabetes distress in type 1 or type 2 diabetes: a precision mental health approach using *n*-of-1 analyses

Brief title: Individual drivers of diabetes distress

Authors:

Dominic Ehrmann*[1,2,3], Norbert Hermanns*[1,2,3,4], Andreas Schmitt[1,3,4], Laura Klinker[1,3,4], Thomas Haak[4], Bernhard Kulzer[1,2,3,4]

* Dominic Ehrmann and Norbert Hermanns share first authorship.

Affiliations:

[1] Research Institute Diabetes Academy Mergentheim (FIDAM), Bad Mergentheim, Germany

[2] Department of Clinical Psychology and Psychotherapy, University of Bamberg, Bamberg, Germany

[3] German Centre for Diabetes Research (DZD), München-Neuherberg, Germany

[4] Diabetes Clinic, Diabetes Centre Mergentheim (DZM), Bad Mergentheim, Germany

ORCID:

Dominic Ehrmann: 0000-0002-5794-5596

Norbert Hermanns: 0000-0002-2903-2677

Andreas Schmitt: 0000-0002-5913-1457

Laura Klinker: 0000-0002-9484-3257

Bernhard Kulzer: 0000-0001-9120-4479

Co-corresponding authors:

Dominic Ehrmann, Email: dominic.ehrmann@uni-bamberg.de

Norbert Hermanns, Email: norbert.hermanns@uni-bamberg.de

ESM Table 1. Zero-order correlations of subjective perceptions of glucose and objectively measured glucose control.

	Hypoglycaemia burden	Hyperglycaemia burden	Glucose variability burden	% < 3.9 mmol/l	% > 10 mmol/l
Hypoglycaemia burden	-				
Hyperglycaemia burden	0.063	-			
Glucose variability burden	0.319	0.578	-		
% < 3.9 mmol/l	0.311	-0.081	0.040	-	
% > 10 mmol/l	-0.052	0.480	0.232	-0.169	-
Glucose CV	0.295	0.160	0.183	0.451	0.128

Correlations based on all observations across participants. Significance level not applicable due to inflation in power because of the nested design. CV = coefficient of variation

ESM Table 2. Separate analyses for type 1 and type 2 diabetes regarding subjective perceptions versus objectively measured glucose control as drivers of daily diabetes distress.

	Type 1 diabetes				Type 2 diabetes			
	**Estimate**	**Standard error**	**t value**	**p-level**	**Estimate**	**Standard error**	**t value**	**p-level**
Subjective perceptions of glucose control as contributors to diabetes distress								
Hypoglycaemia burden	0.077	0.014	5.525	< 0.0001	0.022	0.017	1.247	0.213
Hyperglycaemia burden	0.175	0.014	12.301	< 0.0001	0.106	0.015	7.025	< 0.0001
Glucose variability burden	0.147	0.017	8.787	< 0.0001	0.206	0.018	11.673	< 0.0001
Objective CGM-based markers of glucose control as contributors to diabetes distress								
% < 3.9 mmol/l	0.087	0.046	1.877	0.061	0.036	0.074	0.486	0.627
% > 10 mmol/l	0.058	0.015	3.818	0.0001	0.047	0.021	2.187	0.029
Glucose CV	-0.008	0.032	0.260	0.795	-0.043	0.041	1.049	0.294

Controlled for gender, prior CGM use, number of late complications, and study day. Estimates from the Bayesian mixed-effects linear regression analysis with participant as nested variable. Dependent variable was daily diabetes distress. Independent variables were included simultaneously in the model and defined as random effects. CV = coefficient of variation.

ESM Table 3. Separate analyses for people with vs. without prior CGM use regarding subjective perceptions versus objectively measured glucose control as drivers of daily diabetes distress.

	**With prior CGM use**				**Without prior CGM use**			
	**Estimate**	**Standard error**	**t value**	**p-level**	**Estimate**	**Standard error**	**t value**	**p-level**
Subjective perceptions of glucose control as contributors to diabetes distress								
Hypoglycaemia burden	0.056	0.013	4.225	< 0.0001	0.052	0.019	2.691	0.0072
Hyperglycaemia burden	0.150	0.013	11.533	< 0.0001	0.130	0.018	7.276	< 0.0001
Glucose variability burden	0.171	0.016	10.898	< 0.0001	0.183	0.019	9.480	< 0.0001
Objective CGM-based markers of glucose control as contributors to diabetes distress								
% < 3.9 mmol/l	0.058	0.046	1.266	0.206	0.196	0.068	2.891	0.0039
% > 10 mmol/l	0.041	0.014	2.995	0.0028	0.083	0.029	2.860	0.0043
Glucose CV	-0.0007	0.029	0.023	0.982	-0.092	0.052	1.755	0.079

Controlled for gender, type of diabetes, number of late complications, and study day. Estimates from the Bayesian mixed-effects linear regression analysis with participant as nested variable. Dependent variable was daily diabetes distress. Independent variables were included simultaneously in the model and defined as random effects. CV = coefficient of variation.

ESM Table 4. Impact of individual associations of subjective perceptions and objective CGM metrics of glucose control on psychosocial and glucose parameters at follow-up for people with type 1 diabetes.

Variable at follow-up	Subjective perceptions as contributors to diabetes distress			Objective CGM metrics as contributors to diabetes distress		
	Hypoglycaemia burden	Hyperglycaemia burden	Variability burden	% < 3.9 mmol/l	% > 10 mmol/l	Glucose CV
Depressive symptoms	0.04	0.29	0.25**	-0.13	-0.15	-0.24
Diabetes distress	-0.02	0.41**	0.38***	-0.35***	-0.23	-0.11
Fear of hypoglycaemia	0.15	0.11	0.42***	-0.35***	-0.10	0.10
Fear of complications	0.08	0.22	0.25*	-0.27*	-0.13	-0.05
Diabetes self-management	0.01	-0.11	-0.19	0.14	-0.07	-0.11
Diabetes acceptance	-0.05	-0.03	-0.39***	0.35***	-0.07	-0.15
HbA_{1c}	-0.05	0.37*	-0.01	0.07	-0.15	-0.20
Time in hypoglycaemia (< 3.9 mmol/l)	-0.07	-0.12	-0.04	0.01	0.02	0.06
Time in range (3.9-10 mmol/l)	0.10	-0.43*	0.03	-0.16	0.15	0.29
Time in hyperglycaemia (> 10 mmol/l)	-0.07	0.42*	-0.02	0.15	-0.14	-0.28

Data are standardised beta coefficients from linear regression analysis. Controlled for gender and age. * $p < 0.05$, ** $p < 0.01$, *** $p < 0.001$

ESM Table 5. Impact of individual associations of subjective perceptions and objective CGM metrics of glucose control on psychosocial and glucose parameters at follow-up for people with type 2 diabetes.

Variable at follow-up	Subjective perceptions as contributors to diabetes distress			Objective CGM metrics as contributors to diabetes distress		
	Hypoglycaemia burden	Hyperglycaemia burden	Variability burden	% < 3.9 mmol/l	% > 10 mmol/l	Glucose CV
Depressive symptoms	0.02	0.19	0.32**	-0.38**	-0.10	0.03
Diabetes distress	-0.11	0.31*	0.28**	-0.37**	-0.15	-0.07
Fear of hypoglycaemia	-0.01	0.15	0.34***	-0.19	-0.20	-0.01
Fear of complications	-0.12	0.23	0.13	-0.09	-0.20	-0.10
Diabetes self-management	-0.06	-0.10	-0.20	0.19	-0.02	0.11
Diabetes acceptance	-0.02	-0.23	-0.15	0.28*	0.02	0.10
HbA_{1c}	0.03	-0.01	0.03	0.09	0.12	0.10
Time in hypoglycaemia (< 3.9 mmol/l)	0.05	0.17	-0.15	0.13	0.04	-0.36*
Time in range (3.9-10 mmol/l)	0.02	-0.01	-0.11	-0.07	-0.10	-0.08
Time in hyperglycaemia (> 10 mmol/l)	-0.02	-0.002	0.11	0.06	0.09	0.10

Data are standardised beta coefficients from linear regression analysis. Controlled for gender and age. * $p < 0.05$, ** $p < 0.01$, *** $p < 0.001$

ESM Table 6. Impact of the average of subjective perceptions and objective CGM metrics of glucose control on psychosocial and glucose parameters at follow-up.

Variable at follow-up	Average of subjective perceptions of glucose control	Average of objective CGM metrics of glucose control
Depressive symptoms	0.32***	-0.31***
Diabetes distress	0.39***	-0.33***
Fear of hypoglycaemia	0.34***	-0.27***
Fear of complications	0.23***	-0.22***
Diabetes self-management	-0.19***	0.11*
Diabetes acceptance	-0.29***	0.22***
HbA_{1c}	0.07	0.12*
Time in hypoglycaemia (< 3.9 mmol/l)	-0.06	-0.01
Time in range (3.9-10 mmol/l)	-0.09	-0.12*
Time in hyperglycaemia (> 10 mmol/l)	0.10	0.11*

Data are standardised regression coefficients from linear regression analysis. Controlled for gender, age, and type of diabetes. * $p < 0.05$, ** $p < 0.01$, *** $p < 0.001$

ESM Fig. 1. Cumulative distribution of individual associations of subjective perceptions of glucose control (a-c) and objective CGM metrics of glucose control (d-f) with daily diabetes distress.

a)

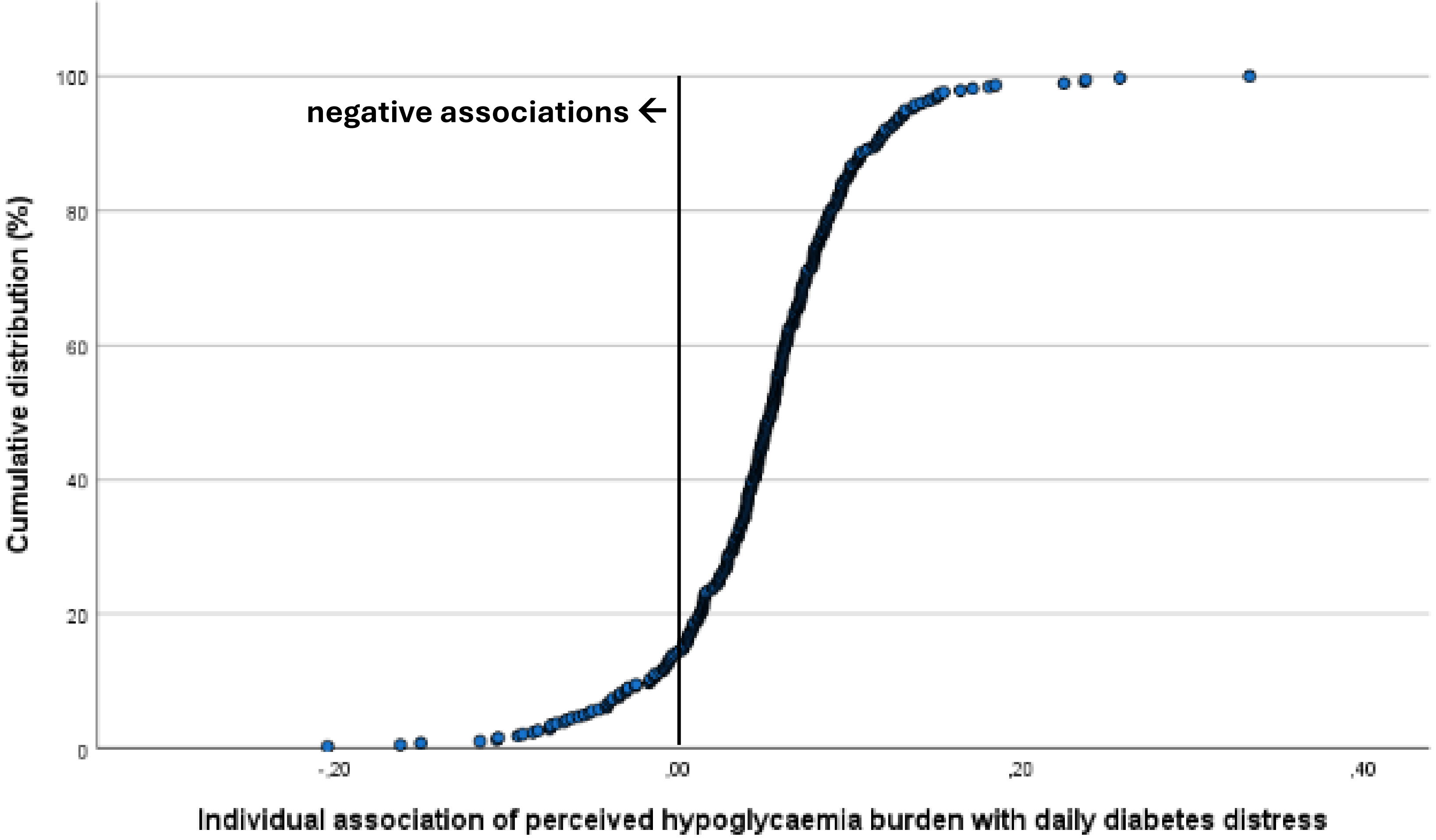

ESM Fig. 1. Cumulative distribution of individual associations of subjective perceptions of glucose control (a-c) and objective CGM metrics of glucose control (d-f) with daily diabetes distress.

b)

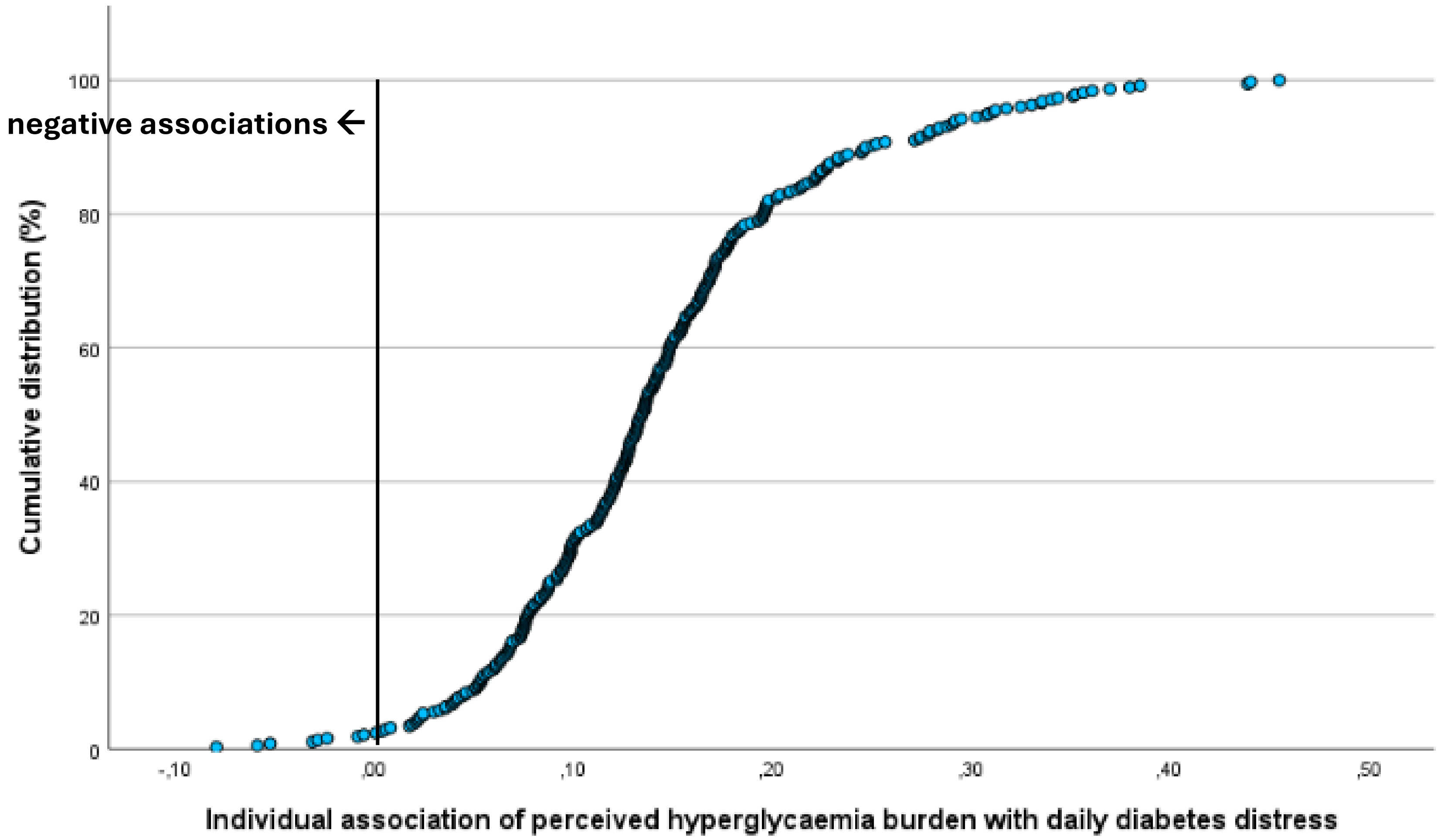

ESM Fig. 1. Cumulative distribution of individual associations of subjective perceptions of glucose control (a-c) and objective CGM metrics of glucose control (d-f) with daily diabetes distress.

c)

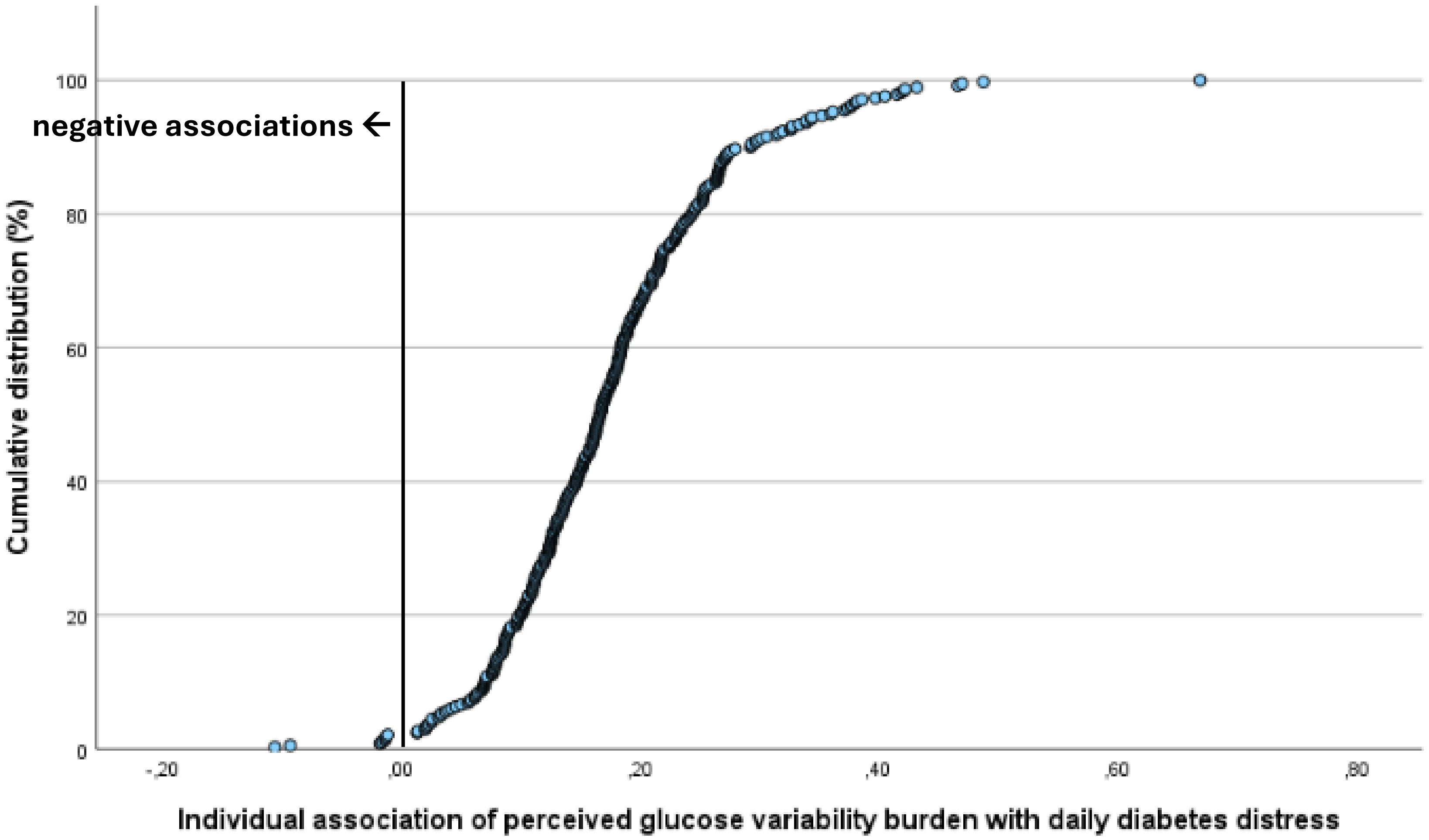

ESM Fig. 1. Cumulative distribution of individual associations of subjective perceptions of glucose control (a-c) and objective CGM metrics of glucose control (d-f) with daily diabetes distress.

d)

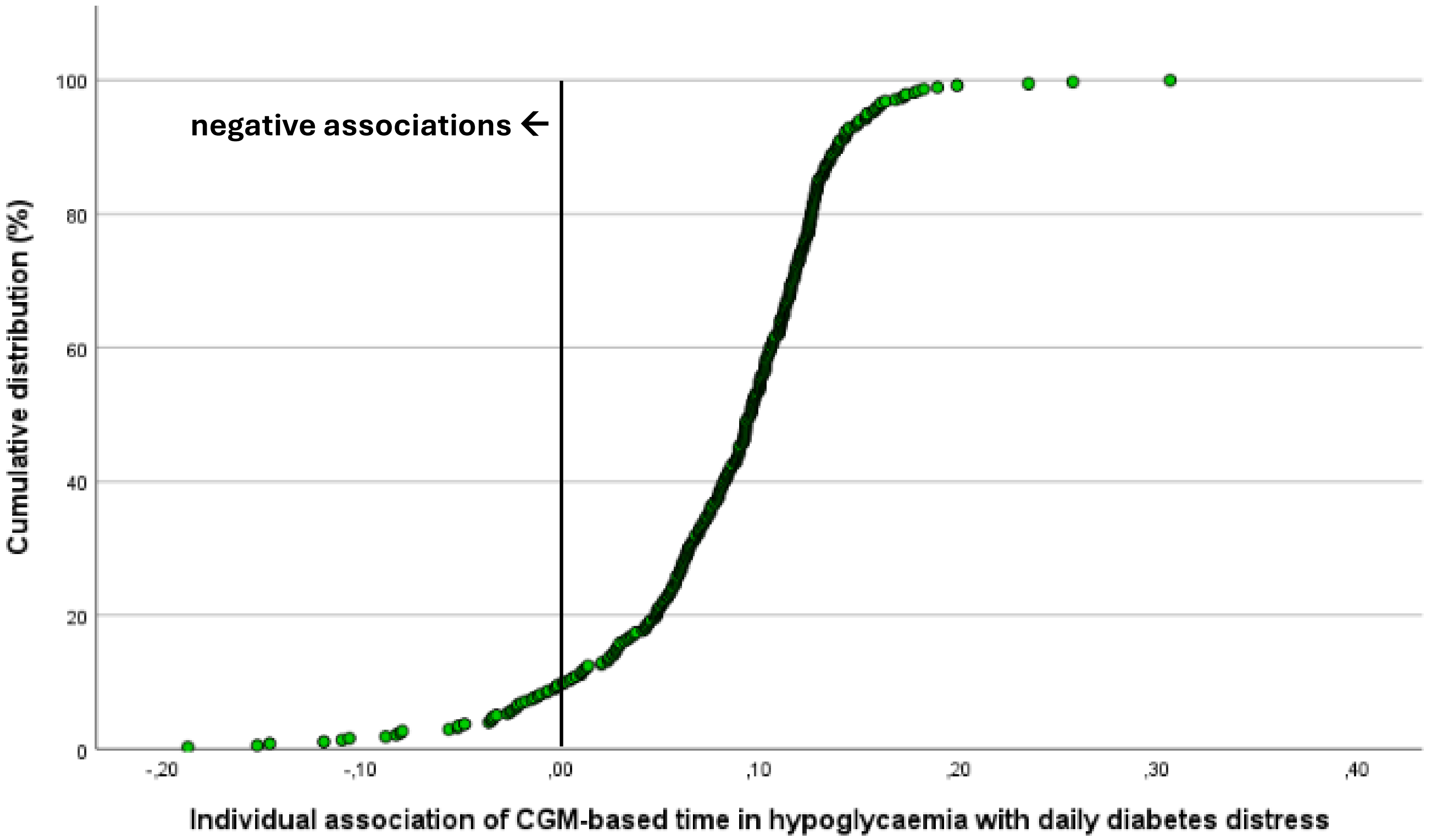

ESM Fig. 1. Cumulative distribution of individual associations of subjective perceptions of glucose control (a-c) and objective CGM metrics of glucose control (d-f) with daily diabetes distress.

e)

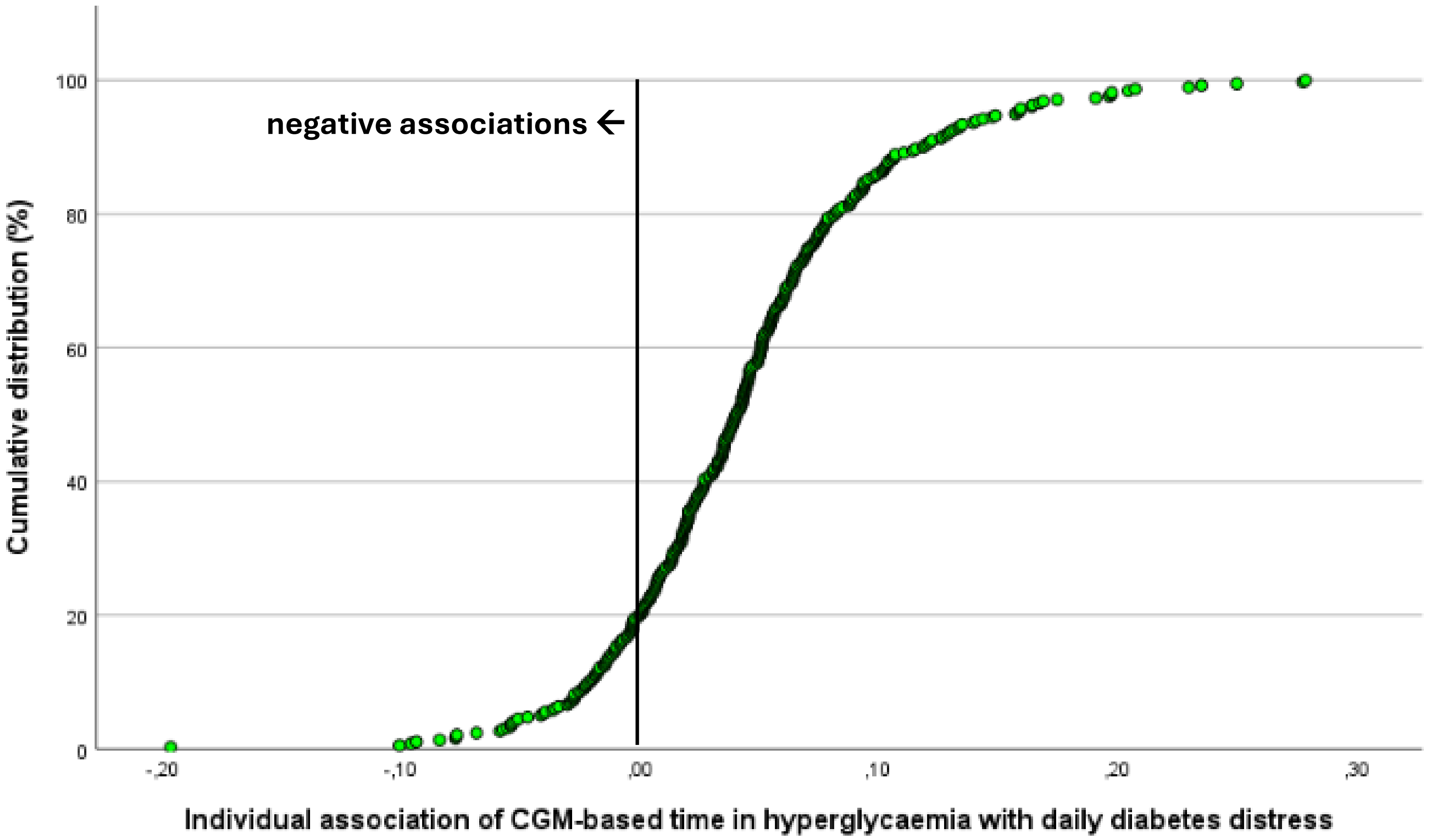

ESM Fig. 1. Cumulative distribution of individual associations of subjective perceptions of glucose control (a-c) and objective CGM metrics of glucose control (d-f) with daily diabetes distress.

f)

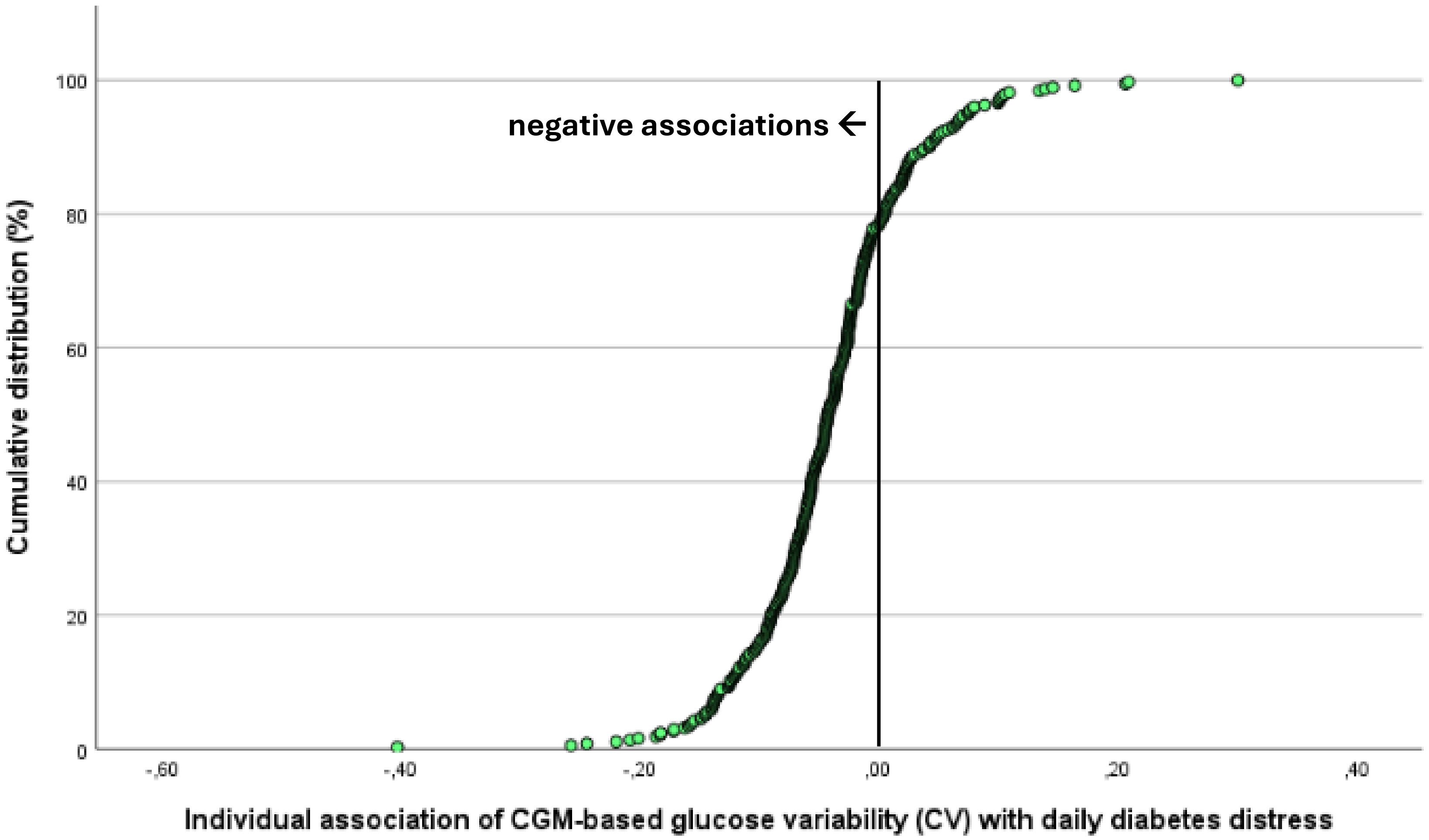

ESM Fig. 2. Impact of the average of subjective perceptions and objective CGM metrics of glucose control on incidence and remission of depressive symptoms. Logistic regression analysis controlled for gender, age, and type of diabetes. Odds ratios in units of standard deviations.

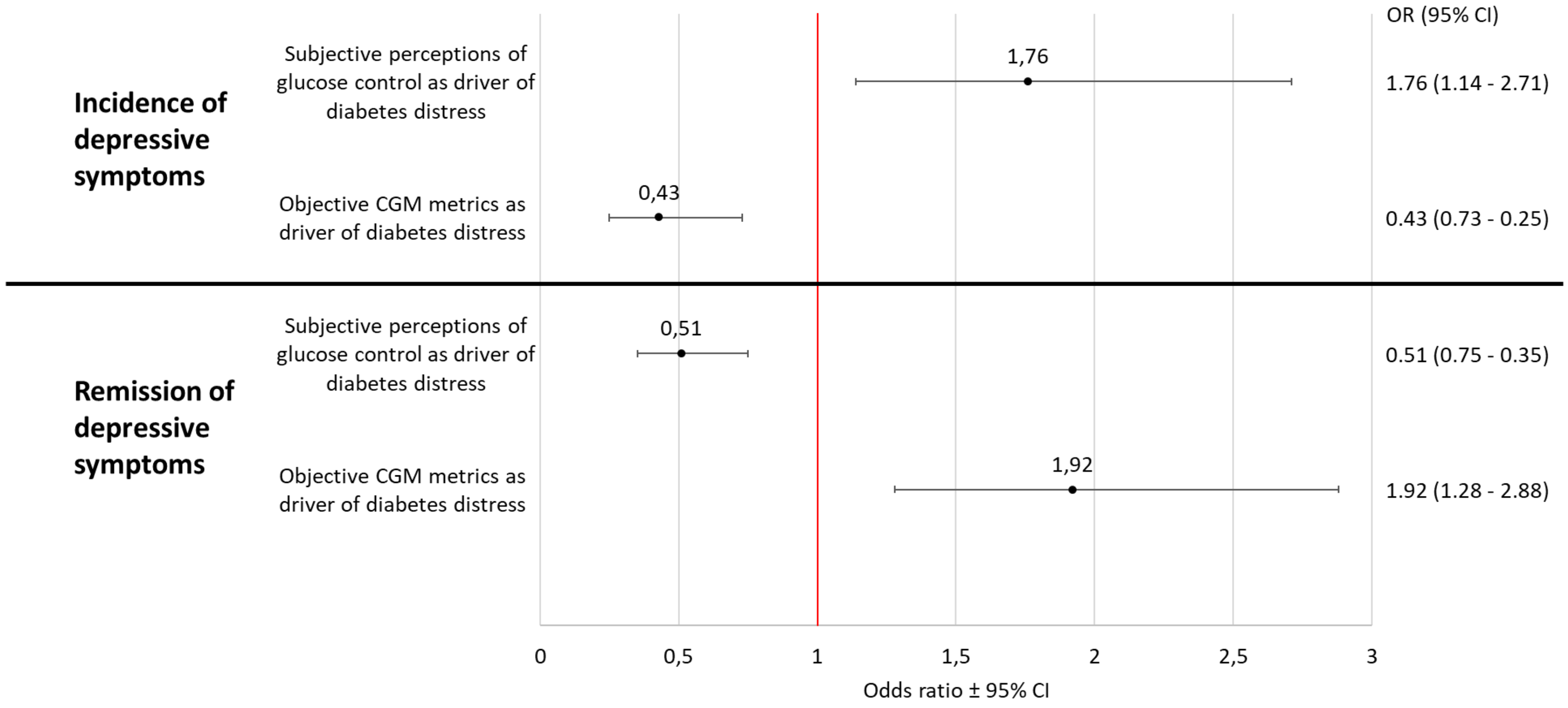

Supplement: Supplementary file 1 — ESM (PDF 589 KB) [file 125_2024_6239_MOESM1_ESM.pdf]
